# Influence of Sample Wetting Method on ESC-Behavior of PMMA under Dynamic Fatigue Crack Propagation

**DOI:** 10.3390/ma15124114

**Published:** 2022-06-09

**Authors:** Tobias Bubmann, Simon Shi, Alexander Brueckner, Teresa Menzel, Holger Ruckdäschel, Alois K. Schlarb, Volker Altstädt

**Affiliations:** 1 Department of Polymer Engineering, University of Bayreuth, Universitätsstraße 30, 95447 Bayreuth, Germany; tobias.bubmann@uni-bayreuth.de (T.B.); alexander.brueckner@uni-bayreuth.de (A.B.); teresa.menzel@uni-bayreuth.de (T.M.); holger.ruckdaeschel@uni-bayreuth.de (H.R.); 2 Chair of Composite Engineering (CCe), Technische Universität Kaiserslautern (TUK), 67663 Kaiserslautern, Germany; simon.shi@mv.uni-kl.de (S.S.); alois.schlarb@mv.uni-kl.de (A.K.S.); 3 Bavarian Polymer Institute and Bayreuth Institute of Macromolecular Research, University of Bayreuth, Universitätsstraße 30, 95447 Bayreuth, Germany; 4 Research Center OPTIMAS, Technische Universität Kaiserslautern (TUK), 67663 Kaiserslautern, Germany; 5 Key Laboratory of Rubber-Plastics, School of Polymer Science and Engineering, Qingdao University of Science and Technology (QUST), Qingdao 266042, China

**Keywords:** PMMA, environmental stress cracking, fatigue, stress cracking agent, da/dN, fatigue crack growth, microplastic

## Abstract

Environmental stress cracking (ESC) is one of the most prominent failure mechanisms for polymer components. The high sensitivity of plastics in regard to environmental influences has always meant that plastics as materials have been viewed very critically in outdoor applications. Recently, the massive occurrence of microplastics in the environment means that questions about the long-term stability of plastic parts and the studies of plastic fragmentation are of great scientific interest. ESC behavior also plays an important role in connection with the formation of microplastics. In this work, the influence of two different sample wetting methods on ESC behavior was investigated. In case A, the sample was in situ wetted with the medium during the measurement by using a sponge. In case B, the sample was wetted by storage in the medium prior to measurement. Different stress cracking agents (SCA) were examined for polymethylmethacrylate (PMMA). Fracture-mechanical fatigue crack propagation (FCP) tests were carried out to quantitatively determine the sensitivity to ESC. Correlations between the absorption behavior and the ESC behavior of the SCA and the resulting morphological phenomena were established. Depending on the wetting method, significant differences in FCP were observed. The in situ wetting of the samples (case A) during the FCP measurement with ethylene glycol (EG) and with deionized water (DI) led to a significant shift in the crack propagation curves to higher ∆K—compared to the PMMA reference. In the case of n-heptane (NH), a more brittle crack propagation behavior was observed due to the chemical interaction with PMMA. The previously immersed samples (case B) give different results. Storage in NH and EG showed no influence on the crack propagation behavior. Samples immersed in DI showed a completely different course of crack growth. At a certain load, a sudden deceleration of the crack propagation and thus a horizontal curve could be seen. Above a certain ∆K value, crack growth began again. Depending on the immersion time (14, 30, or 60 days), this so-called stepped behavior shifted to lower da/dN values.

## 1. Introduction

An important and well-known disadvantage of plastics is their high sensitivity to environmental influences such as acid rain, dilute acids, alkalis, and/or UV irradiation. In addition, atmospheric oxygen has an accelerating effect on degradation. In many cases, cracking starting at the surface of the components is observed, which is associated with a loss of inherent mechanical properties as a function of exposure time. Amorphous plastics are particularly sensitive to the environmental influences described. Specific organic liquids can initiate cracks or hairline cracks, even at the slightest external stresses (often an order of magnitude lower than the actual tensile or actual tensile or flexural strength of the polymer). In some cases, the manufacturing residual stresses of the glassy polymer are already sufficient to initiate failure. The first very comprehensive study was already published in 1996 by D. C. Wright [1]. The studies of that time are of particular importance today when more and more microplastics are being found in the environment. Especially the formation of secondary microplastics can be explained by the stress cracking sensitivity of plastics. However, the storage times in the environment and UV exposure are considerably longer than in the 1996 studies. In any case, fracture mechanics plays an important role in quantitatively describing the kinetics of the fragmentation of plastics into microplastic particles with a size smaller than 5 mm.

Polymethyl methacrylate (PMMA) is one of the most frequently used polymers in applications that require high transparency. The reason is its excellent scratch resistance, high weathering, and UV-light stability as well as a high chemical resistance [2,3]. These properties are particularly important in areas of application where transparency is required, e.g., in the construction sector (greenhouses, terrace roofing with light panels) or in medical technology (e.g., syringes, hoses) [4].

Based on the application, direct contact and steady wetting with various environmental liquids (e.g., water, alcohols, or oils), also known as stress cracking agents (SCA), requires high chemical resistance. The influence of such SCA on the mechanical behavior of a polymer material is referred to as environmental stress cracking (ESC) behavior. ESC is one of the most common causes of shortening the service life of polymer materials. It is responsible for about 25% of all failures of plastic products in service [5]. There is always a risk of stress-induced cracks when a component is exposed to mechanical loads and SCA at the same time. The consequences of such a failure are often severe and can be lifetime determining, especially in medical care [6,7]. The formation of ESC in polymers involves molecular interactions between the polymers and a specific SCA. Although SCAs do not cause chemical degradation of the polymer, they can slowly penetrate the molecular structure of the polymer (under mechanical stress) and disrupt the intermolecular forces binding the polymer chains. This interaction accelerates molecular disentanglement and subsequent macroscopic brittle cracking.

Under everyday conditions, the diffusion of a low-molecular substance can lead to an unexpected brittle fracture of a plastic component [5,7]. From a physical point of view, the diffusion strongly depends on the solubility factors of the SCA. Hansen [8] and Hansen et al. [9] quantitatively describe the ESC phenomenon in a polymer material by correlating the interaction between polymer and solvent with molecular parameters (e.g., the molar volume). They found that the ESC behavior of polymeric materials depends not only on the solubility parameters of the solvent relative to those of the polymeric material but also on the size and shape of the ESC agent. The ESC resistance decreases for low-molecular substances (SCAs) due to their smaller molecular size and less bulky molecular structure. In addition, external factors have a noticeable impact on the ESC resistance of polymers—the physical and chemical properties of the SCA, ambient temperature, type, and speed of the applied load, and especially the wetting conditions [5,10].

The investigation of the ESC behavior under mechanical load can be carried out under static or dynamic load. Mechanical tests of thermoplastics under static loading, either tensile, bending, or creep tests, are widely known in the literature [11,12,13,14,15,16,17,18,19,20]. These investigations were either carried out on specimens previously immersed in the SCA [20] or during in situ wetting [10,17,18]. Arnold [20] studied the effects of the diffusion properties of several SCAs with different solubility parameters and molar volumes on environmental stress crack initiation for PMMA. Depending on the degree of diffusion, testing rate, and SCA, crazing can be delayed in tests with longer pre-immersion.

The examination of the service life of components is usually carried out under dynamic load and practical exposure to a medium. Fatigue crack propagation tests focus on the propagation of a single crack in a suitable specimen. The crack propagation rate (da/dN) is measured as a function of the amplitude of the stress intensity factor (∆K) at the crack tip. This method is one of the most sensitive in mechanical testing to determine the kinetics of crack propagation in a specimen. Furthermore, the method considers the interaction of the crack tip as a function of SCA, residual stresses, or orientations. Experimentally, transparent polymers are favorable because the position of the crack tip can also be tracked microscopically [21,22,23,24,25]. The influence of the test temperature, the crosslinking density, or additives can be precisely determined with high sensitivity. The effects of molecular weight, molecular weight distribution, test frequency, etc., are described by Altstädt in [5,26] for various polymer materials. However, the number of publications on the influence of SCA on thermoplastics under dynamic loading is rather small.

Various exposure scenarios of a specimen to SCA can significantly affect the crack propagation behavior. Depending on the SCA and the polymer material, different behaviors can be observed, depending on the interaction parameters or the diffusion coefficients. To the best of our knowledge, the influence of the wetting process and the solvent on the micromechanical crack propagation behavior has not yet been investigated.

In this work, the effects of the treatment of the test specimen are examined experimentally. The main questions to be answered in the study are (a) how solvents with opposite solubility parameters affect crack propagation in PMMA and (b) how the fatigue crack propagation behavior of PMMA under the influence of an SCA is affected by the selected wetting processes of the sample. A correlation of the solvent properties, the wetting process, and the deformation mechanisms occurring during crack propagation needs to be established.

## 2. Materials and Methods

### 2.1. Materials and Solvents

PMMA (Plexiglas^®^ 8N) (**8N**) was provided by RHÖM (Essen, Germany) with a M_w_ of 77,600 g/mol and M_n_ of 47,300 g/mol. The material was dried at 60 °C for a minimum of 12 h under a vacuum prior to processing.

The SCAs used for the investigation of the ESC behavior were deionized water (**DI**), ethylene glycol (**EG**), and n-heptane (**NH**). EG and NH were purchased from Merck KGaA (Darmstadt, Germany).

### 2.2. Experimental

#### 2.2.1. Sample Preparation

Plate-like test specimens with dimensions of 70 mm × 70 mm × 4 mm were prepared by injection molding (Arburg 470 H 1000-170, Loßburg, Germany) with an injection pressure of 100 bars. The temperature of the melt was set at 270 °C. The mold temperature was 60 °C. From the injection molded plates, different samples were prepared by means of sawing: Compact tension (CT)-specimen was according to ISO 15850/ASTM E647 for the fatigue crack growth tests and bars had dimensions of 12 mm × 36 mm × 4 mm for the absorption investigations.

#### 2.2.2. Adsorption Investigations

To get an impression of the interaction between the polymer and the fluid, absorption measurements were conducted at room temperature (23 °C) and elevated temperature (48 °C). Samples with dimensions of 12 mm × 36 mm × 4 mm were weighed prior to the submersion in the pure SCA, with no dissolution. After certain intervals, the specimens were taken out, wiped dry, weighed, and submerged again.

Samples for the fatigue crack growth experiments via the method prior immersion (st) were stored, depending on the solvent, for 15, 30, and 60 d. Here the prepared CT samples with an initial 1 mm sharp pre-crack were used. The abbreviation, e.g., for the 30 d prior immersed sample is 8N/st/30. “St” here means “stored”.

#### 2.2.3. Fatigue Crack Propagation (FCP) Measurements

**Figure 1:**
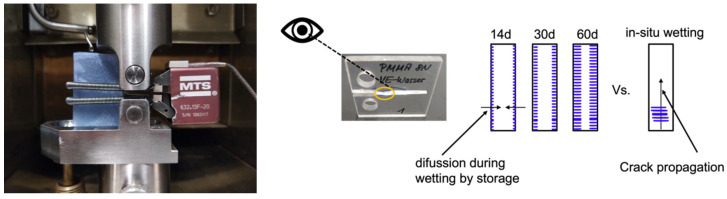
Experimental setup for the ESC measurements under fatigue loading and in situ wetting. Scheme of the SCA diffusion during storage compared to crack propagation.

Fatigue crack propagation tests were conducted on CT specimens. Tests were performed according to ISO 15850/ASTM E647 at 23 °C and 50% relative humidity. One measurement was done with neat PMMA at 50 °C and 50% relative humidity. A frequency of 10 Hz was chosen. Furthermore, the controller was in a load control mode with an R ratio of 0.1 using a servo-hydraulic testing machine (IST Hydro Pulse MHF, Zwick, Germany). The amplitude of the cyclic stress intensity factor (ΔK = K_max_ − K_min_) as a function of crack length was controlled by software. This ensured a defined increase of ΔK with the number of load cycles. Before the measurement, a 1 mm sharp pre-crack was first introduced in the V-notch of the sample with a sharp razor blade. The crack opening displacement (COD) was measured with a crack opening transducer (632.13F-20, MTS, Sensor Technology GmbH & Co. KG, Lüdenscheid, Germany), which was fixed symmetrically to the plane of the crack on the front face of the CT specimen (Figure 1). From the COD of the crack and the associated force, it was possible to continuously determine the compliance and, from this, the crack length as a function of the numbers of cycles using equations from Saxena and Hudak [27] Each measurement was repeated at least twice to minimize experimental error. For in situ wetting, the samples were covered with a sponge on both sides, continuously soaked with the SCAs, DI and NH. This ensured that the sample and in particular the tip of the crack was completely wetted with the SCA throughout the measurement.

#### 2.2.4. Scanning Electron Microscope (SEM)

The fracture surfaces of the CT-specimens after testing were analyzed with a scanning electron microscope (SEM) from JEOL-JSM-6510 from JEOL (Freising, Germany). The acceleration voltage was set to 10 kV and the samples were sputtered with a 1.3 nm thick platinum layer prior to measurement.

## 3. Results and Discussion

### 3.1. Absorption Measurements

The comparison of the Hansen solubility parameters of the polymer material and the solvent already allows an assessment of the interaction and aggressiveness of the solvent to the polymer. In particular, the ESC behavior depends on the size of the solvent molecules and the molar volume of the SCA which depends on the molecular structure. The smaller the size of the solvent molecule, the better the solvent penetrates into the polymer material. The more similar the Hansen solubility parameters of solvent and polymer are, the more aggressive the solvent. The Hansen and Hildebrand solubility parameters of PMMA and the SCAs used, and their molar volumes are listed in Table 1.

Comparing only the molar volume of the SCA, DI is the solvent with the lowest molar volume and thus the highest diffusion rate in PMMA. Therefore, EG and NH should be absorbed more slowly.

In contrast to the molar volume, NH comes closest to PMMA with 15.3 when comparing the total Hildebrand parameter δ_T_ of the solvents with the value of PMMA (20.0). This indicates that NH is more aggressive compared to DI and EG as it has higher chemical compatibility. Therefore, NH may dissolve PMMA faster over time or at elevated temperatures.

**Figure 2:**
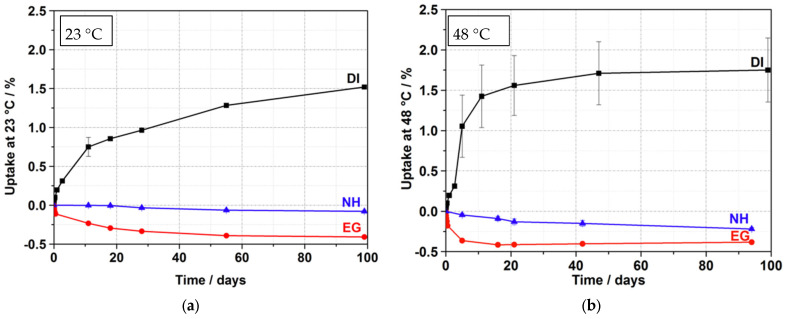
Absorption properties of PMMA stored at 23 °C (**a**) and 48 °C (**b**) in DI, NH, and EG.

To get a first insight into the absorption properties of DI, NH, and EG in PMMA, absorption tests were performed. The uptake of the SCA was measured by the increase in the weight of the samples. The results are shown in Figure 2 at 23 °C (a) and 48 °C (b).

None of the SCAs led to a strong dissolution of PMMA at 23 °C or 48 °C. Thus, all solvents can be used as SCA for the ESC measurements under dynamic loading. The absorption curve for DI is approximately Fickian, with a weight gain after 100 d of 1.5% at 23 °C (a). This is the expected behavior for a liquid with limited chemical compatibility but small molecular size [20]. Swelling of the material was not observed. The investigations at 48 °C (b) showed that the diffusion takes place at a higher speed and that a plateau is reached after about 50 d. The diffusion rate in PMMA is therefore much higher compared to lower temperatures. A possible explanation regarding the large error bar is the strong diffusion rate on one side and the rapid increase in interaction between fluid and polymer. The possible movement allows for water to diffuse into the polymer but also out of the polymer. The steady increase in weight achieved in 100 days at room temperature is achieved 10 times faster.

Compared to DI, the diffusion behavior for EG and NH is completely different regardless of temperature. Despite the strong difference in molar volume—the value for EG is much smaller than the value for NH—and δ_T_ (NH is much closer to the value of PMMA than EG), both SCAs initially showed only a small weight loss over the measurement time (less than 0.5%). This could be due to the solubility of mobile components in PMMA. This effect was previously described in the literature [19]. From the results shown, it can be concluded that there is little or no interaction of the solvent with PMMA. Fracture-mechanical fatigue crack propagation tests, in which diffusion plays a minor role, can help to investigate the influence on the mechanical properties in more detail.

### 3.2. Fatigue Crack Growth Experiments

**Figure 3:**
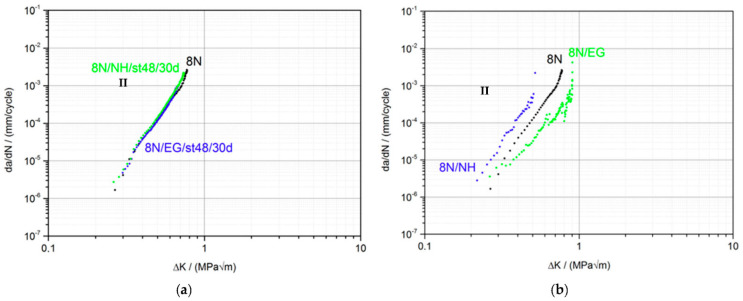
FCP-behavior of PMMA prior to immersion (**a**) and in situ wetting (**b**) in EG and NH.

For the second part, the influence of SCA on the mechanical behavior of PMMA in the fatigue crack propagation test was investigated. The focus was on how the SCA and the wetting process affect crack propagation. This was assessed by using the characteristic values: ΔK_th_, the slope of the FCP curve in area II, and Kc_max_. Figure 3 shows the FCP measurements for the pure PMMA without medium and the ESC behavior for the type of wetting, (a) previous immersion of the CT sample with crack for 30 d at 48 °C and in situ wetting, (b) the entire sample up to the crack tip.

When comparing the crack propagation curve of the pure PMMA material and the samples previously immersed in EG (blue curve) and NH (green curve) (a), no influence can be seen after 30 d at 48 °C storage condition. All three curves are almost congruent. The characteristic values ΔK_th_, steepness of the curve and Kc_max_ are identical within the measurement accuracy (Table 2). The analysis of the fracture surfaces of the specimens by SEM support our findings (Table 3). The fracture surfaces of the pure PMMA and the previously stored samples with EG and NH all look identical. These results are somewhat surprising since FCP measurements are very sensitive and can detect minimal differences in crack propagation behavior. From this it can be concluded that there is no influence of EG and NH on the crack propagation behavior under these storage conditions. Even the crack of about 1 mm in the CT sample does not accelerate the diffusion of SCA into the sample or to the crack tip. When comparing the fracture surfaces of the pure PMMA and the samples 8N/NH/st48/30d and 8N/EG/st48/30d previously stored in EG or NH, no differences are discernible. All surfaces are structureless and smooth, as is usually the case with a brittle material such as PMMA.

A possible reason for the fact that no influence was visible may be due to the way the samples were stored in the medium. Only a layer close to the surface was saturated with the medium. No medium evidently penetrated through the crack into the crack tip running transversely to the surface, since the crack apparently closed again due to relaxation to such an extent that the capillary forces were not sufficient to transport the medium into the volume in front of the crack tip by diffusion. These results are in contrast to the results of Arnold [19]. He found a strong decrease in the elongation at break and thus in the embrittlement in tensile tests on samples that were only immersed in EG for 1 min. In the tensile test, the surface has a completely different meaning than in the CT sample in the FCP test. Here the crack runs across the volume of the sample.

**Figure 4:**
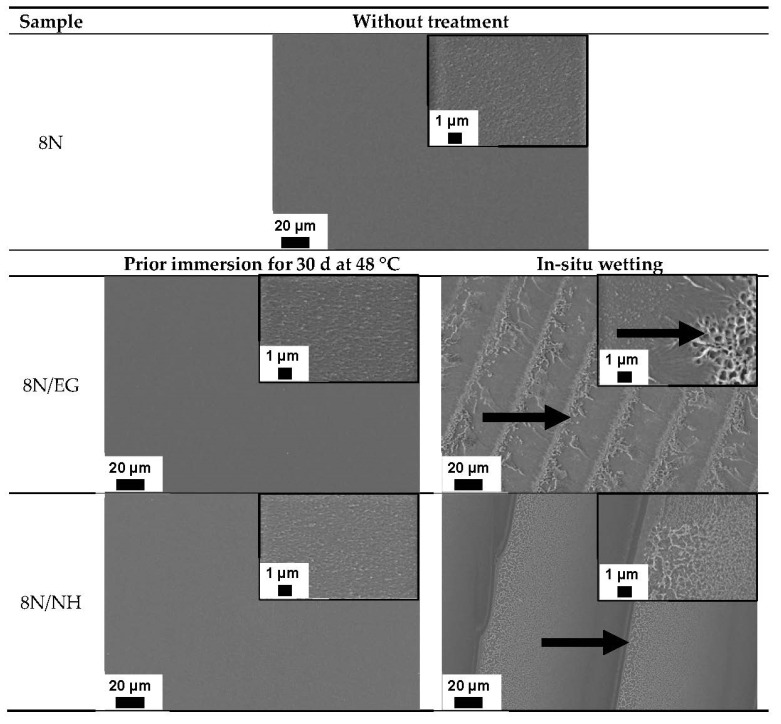
Comparisons of the fracture surfaces of the 8N samples after immersion and in situ wetting via SEM images of the region II (see Figure 3).

The FCP measurements show a completely different behavior when the samples are wetted in situ with EG and NH (b). Continuous wetting of the sample and thus the crack tip with EG leads to the same ΔK_th_ as with pure PMMA. However, the crack growth accelerates more slowly, as shown by the lower gradient in region II (Table 2). The water that has diffused into the crack tip leads to a softening effect and thus to a delayed unraveling of the molecular chains. This is associated with a more ductile crack propagation behavior. This also results in an increase in Kc_max_ from 0.82 MPa√m for neat PMMA 8N to Kc_max_ 0.99 MPa√m for the EG treated sample. In addition, the experimental scatter of the measurement points is significantly higher at higher K values in the samples wetted with SCAs. This may be due to the fact that the solvent has less time at higher da/dN values to diffuse into the crack tip and interact with the PMMA material there. Thus, the crack is stopped by EG or proceeds normally as in the pure material (lamination). Here, too, the molar volume and the chemical nature play an important role. Because there is no diffusion into the material, the SCA can only act while being squeezed into the crack tip while the crack tip is dynamically (10 Hz) opening and closing. The influence described is clearly visible in Figure 4. Starting from the edge of each lamella in the direction of crack propagation, ramps and threads appear due to plastic deformation (marked by arrows).

The medium NH causes a fundamentally more brittle fatigue crack propagation behavior compared to EG and compared to pure PMMA. The characteristic values show a shift of the crack propagation curve to the left (Table 2). This embrittlement is due to a lower solubility according to Hansen and Hilde. The fracture surfaces of the NH-wetted sample have a much finer striation pattern and are therefore less prone to crack propagation. As in EG, striation formation can also be observed in NH, but the distance is much wider.

The factors responsible for the different behavior of the two wetting methods in the fatigue crack propagation test are, on the one hand, the lower diffusion rate of the medium and, on the other hand, the chemical interaction of EG and NH in the PMMA. Previous wetting of the CT sample despite the presence of a crack has no direct influence on the micromechanical behavior under dynamic loading. The in situ wetting process allows the SCA to act directly at the crack tip, where it can directly influence crack propagation behavior, which is reflected in a more brittle or more ductile material behavior. The physical and chemical nature of the SCA plays a decisive role in the mode of action of the solvent.

**Figure 5:**
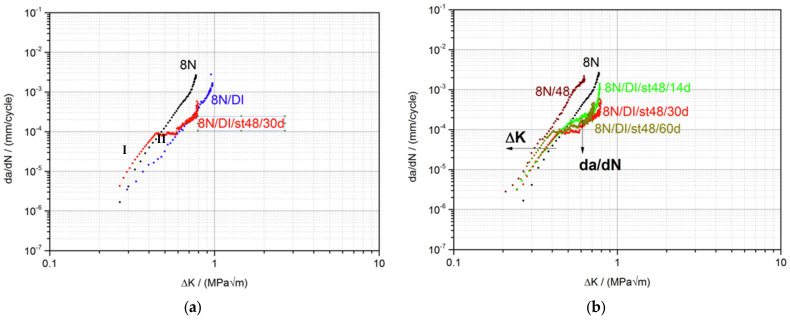
ESC behavior of PMMA after prior immersion and in situ wetting in DI (**a**) and influence of immersion time in DI on the fatigue crack growth behavior of PMMA (**b**).

An interesting behavior was exhibited by DI in conjunction with PMMA. Figure 5a shows the fatigue crack propagation behavior between the in situ wetted sample and the sample previously immersed for 30 days at 48 °C prior to testing. In (b) the comparison of the influence of different storage times on the dynamic-mechanical behavior is shown.

**Figure 6:**
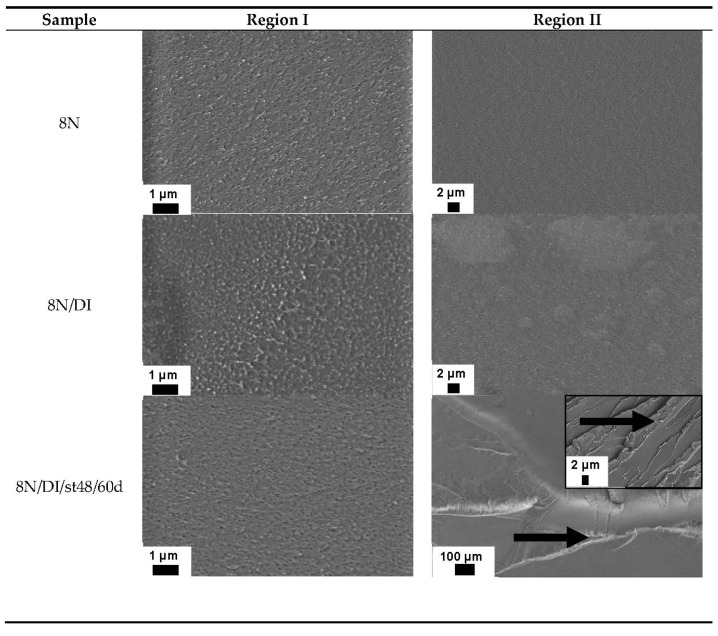
Comparisons of the fracture surfaces of the 8N samples after in situ wetting and prior immersion via SEM images of region I and II (marked in Figure 5).

In situ wetting of PMMA with DI (Figure 5a) leads to the same results as with EG. A shift of the entire curve to the right implies a more ductile behavior with the same slope of the crack propagation curve in region II. Compared to the large influence on the crack propagation behavior, there are no major differences at the microscopic level visible (SEM images shown in Figure 6). In comparison to NH and EG, no formation of stirrup is visible in area II. This could be due to the accelerated diffusion of DI in the PMMA. It can therefore be assumed that the crack tip is always slightly wetted with DI, regardless of whether the sample is loaded or unloaded.

The crack propagation behavior of CT samples stored 30 days in DI at 48 °C differs significantly from the samples with in situ wetting. Up to a ΔK of 0.44 MPa√m, the curve is as usual. With an increase in ΔK, the crack propagation rate, da/dN, also increases. With a higher ΔK, the crack propagation rate remains almost constant at a value of 10^−4^ mm/cycle in the double logarithmic scale, regardless of the ΔK value. Below ΔK of 0.44 MPa√m, the crack propagation curve is similar to that of pure PMMA. This is also reflected in the comparison of the region I fracture surfaces of the two CT-specimens in the SEM (Figure 6). When this plateau is reached with slope = 0, the crack grows at a constant propagation rate despite an increase in ΔK (slope = 0). This plateau suggests that it is caused by DI diffusing into the crack tip. In this load range, this can lead to the crack in the PMMA growing as a result of the macromolecules continuously disentangling as a result of the penetration of the medium and the associated higher molecular mobility. This slower process of disentanglement is only possible in this low crack propagation range. Above a ΔK value of about 0.6 MPa√m, regular crack growth begins again, which is then presumably dominated by chain scission. Then, the crack proceeds in the same way and identically as in situ wetted samples. However, Kc_max_ decreases from 1.03 MPa√m for the in situ wetted sample to 0.86 MPa√m, which is the same as for pristine PMMA.

This decrease in Kc_max_ could be a consequence of a lower local temperature at the crack tip. Comparing the curves of 8N with those of 8N/48, please consider that the PMMA was measured under temperature, a shift in the curve and therefore in Kc_max_ can be seen at the same level as the change for the samples with the two different wetting methods.

The plateau is also clearly visible on the fracture surface of the PMMA sample in area II (Figure 6). The fracture surfaces in area I of the pure PMMA and the stored sample (8N/DI/st48/30d) are very similar. Upon reaching region II, a visible change in the appearance of the fracture surface can be seen. Compared to the still quite smooth surface of pure PMMA, the sample stored in DI shows plastic deformation due to the formation of striations. Due to the high plastic deformation, so-called humps appear at the edges of the ramp, which give the ramp edge a bead-like thickening. In addition, the formation of crazing can be observed. As previously described, this is evidence of a significant slowdown in the propagation rate.

In a further experiment, the influence of the storage time on the plateau in the propagation rate was examined. Crack propagation curves are presented for the samples stored in DI at 48 °C for 14, 30 and 60 days (Figure 5b) together with the pure PMMA and the PMMA with in situ wetting.

The difference is that with increasing storage time in the medium and thus longer time for the diffusion of DI into the sample, the da/dN value at the plateau decreases (values in Table 3). This can be attributed to the diffusion depth and the formation of the plastic zone in front of the crack tip. The plastic zone increases in diameter at higher loads and, at a certain load, hits the volume in the PMMA that has already been penetrated with DI. Below this load, the plastic zone only spreads in the non-wetted part of the PMMA sample. This is also reflected in the fracture surfaces of this plateau at the different immersion times.

Another difference lies in the fact that a shift in the overall curves is observed with longer storage times. This effect is best observed below the plateau where DI has no effect on crack propagation behavior. Comparing the curve below the plateau, the shift leads to the values of PMMA measured under temperature (brown curve of sample 8N/48). Here, the temperature leads to slight embrittlement, which can be clearly seen in the SEM images of area I in Figure 6. Compared to the pure PMMA sample without temperature and SCA treatment, a smoother fracture surface is observed in the dipped samples under temperature. This served as evidence of why a left shift is visible. This does not come from the SCA but from temperature storage, which is shown by the measurement under temperature.

The formation of a plateau with a constant crack propagation rate that is independent of ∆K only occurs in tests with storage in DI. In this case, there is a complex interplay between the diffusion depth of the medium and the geometric formation of the plastic zone around the crack tip. The longer the immersion time in DI, the deeper the medium has already penetrated through the surface into the sample. Due to the physics of Fick’s law, it is not possible to saturate the entire sample (thickness 4 mm) with DI in a realistic experimental time. Therefore, the stored samples are still dry in the core region. The amplitude of the stress intensity factor ΔK at the crack tip creates a plastic deformation zone. With increasing stress intensity ΔK, the zone increases in volume. At a certain stress intensity ΔK, the formation of the plastic zone is strongly influenced by the medium in the surface layer of the specimen—perpendicular to the crack propagation direction (see arrow in Figure 6 sample 8N/DI/st48/60d). The medium in the surface layer has an additional plasticizing effect, so that an increase in the stress intensity is compensated for by further volume work in the form of disentanglement phenomena in the surface layer penetrated by the medium. Above a certain threshold, this causes only a very small increase in the crack propagation rate (da/dN) (plateau area). If ΔK increases further, the crack propagation is accelerated again, since micromechanically, it is no longer the disentanglement processes of the macromolecules which dominate the crack propagation, but rather chain fracture. This means that in a certain ΔK area, particularly the penetrating medium delays the crack initiation. The ΔK level that has to be reached for the penetrating medium to influence crack growth depends strongly on the storage time at 48 °C.

## 4. Conclusions

In this study, the ESC behavior of PMMA under the influence of different media that trigger environmental stress cracking (DI, EG, and NH) was investigated using fracture-mechanical fatigue crack propagation tests. The influences of in situ wetting in case A or in case B storage in a medium for a certain time, on the crack propagation behavior were analyzed. Interesting differences were observed depending on the wetting process. Especially the formation of secondary microplastics can be explained by the stress cracking sensitivity of plastics. However, the storage times in the environment and UV exposure are considerably longer than in these studies. In any case, fracture mechanics plays an important role in quantitatively describing the kinetics of the fragmentation of plastics into microplastic particles with a size smaller than 5 mm.

PMMA samples previously immersed in EG and NH for 30 d at 48 °C showed no effect on fatigue crack propagation behavior, despite the strong differences in molar volume and chemical structure. With in situ wetting of the crack tip with EG during the FCP measurement, a more ductile crack propagation behavior and with NH a more brittle crack propagation behavior, occur compared to untreated PMMA samples. With in situ wetting, DI shows the same spreading behavior as EG. However, previously immersed samples show a completely different behavior. Depending on the storage time in the medium, the wetted area in PMMA Sample I progressed differently in the depth of the sample. Depending on the penetration depth, the water has a more or less strong influence on the crack propagation through the volume of the sample. With increasing penetration depth, the dry core loses its influence on crack propagation. Above a certain penetration depth, phenomena occur where crack propagation occurs discontinuously with abrupt stops or moves to plateaus with a constant crack propagation rate. These phenomena occur at a certain da/dN value, which in turn depends on the wetting time. When a certain ΔK value is reached, the crack runs again as in the in situ wetted sample. The phenomena described here can be important for molded plastic parts that are exposed to liquids and changing loads. Plastic parts lying on the beach, for example, are exposed to periodic stresses caused by the waves, which lead them to break up piece by piece and thus contribute to the formation of microplastics. In this way, it can be estimated how sensitively a certain plastic behaves under certain application-relevant conditions in the presence of an individual liquid substance under dynamic loading.

## Figures and Tables

**Table 1:** Hansen and Hildebrand solubility parameters of PMMA and SCAs and their molar volumes (values selected from data listed in [1,2]).

Solvent/ Polymer	Molar Volume /cm^3^/mol	$\mathrm{Dispersive}\;\delta_{D}$ /MPa^0.5^	$\mathrm{Polar}\;\delta_{P}$ /MPa^0.5^	$\mathrm{Hydrogen}\;\mathrm{Bonding}\;\delta_{D}$ /MPa^0.5^	$\mathrm{Total}\;\delta_{T}$ /MPa^0.5^
NH	147.4	15.3	0.0	0.0	15.3
EG	55.8	17.0	11.0	26.0	32.9
DI	18.0	15.5	16.0	42.4	47.9
PMMA	-	17.7	6.7	6.2	20.0

**Table 2:** Determined values from the FCP measurements under ESC for EG and NH via the two wetting procedures and the neat PMMA material.

Sample	∆K_th_/MPa√m	Slope	Kc_max_/MPa√m
8N	0.27	5.7	0.82
8N/NH/st48/30d	0.26	6.3	0.80
8N/EG/st48/30d	0.30	6.2	0.73
8N/NH	0.22	5.9	0.71
8N/EG	0.26	4.4	0.99

**Table 3:** Determined values (∆K_Threshold_ and Kc_max_) from the dynamic fatigue measurements under ESC for DI via the two wetting procedures and the neat PMMA material.

Sample	∆K_th_ /MPa√m	∆K_start_ of Plateau /MPa√m	da/dN_start_ of the Plateau /mm/Cycle	Kc_max_/ MPa√m
8N	0.27	-	-	0.82
8N/DI	0.29	-	-	1.03
8N/DI/st48/14d	0.24	~0.52	1.3 × 10^−4^	0.88
8N/NH/st48/30d	0.27	~0.44	9.0 × 10^−5^	0.86
8N/EG/st48/60d	0.24	~0.40	8.9 × 10^−5^	0.80

## Data Availability

The exact data sets of the generated and shown results will be gladly provided upon request.
